# A case report of bilateral persistent sciatic artery: Bilateral aneurysm with thromboembolism of the right lower extremity

**DOI:** 10.1097/MD.0000000000039125

**Published:** 2024-09-13

**Authors:** Yi Mao, Li Chen, Zhi-Xing Liu

**Affiliations:** a Department of Ultrasonography, The First Affiliated Hospital of Nanchang University, Nanchang, China; b Department of Ultrasonography, Ganjiang New District Peoples Hospital, Nanchang, China.

**Keywords:** bilateral persistent sciatic artery, thromboembolism, ultrasound diagnosis, vascular aneurysm.

## Abstract

**Rationale:**

Persistent sciatic artery (PSA) is a rare congenital vascular anomaly. The sciatic artery, which normally regresses to become the inferior gluteal artery during fetal development, persists as a direct branch of the internal iliac artery.

**Patient concern:**

We report a 78-year-old female who was admitted due to sudden pain, numbness, and loss of sensation in the right lower limb.

**Diagnoses:**

Acute thromboembolism in the right leg, bilateral PSA, and bilateral aneurysm.

**Interventions:**

After the super-selective embolization, lower limb arterial thrombolysis treatment was performed. After symptom relief, a computed tomography angiography was conducted to clarify the vascular variations.

**Outcomes:**

After relief of lower limb embolism, long-term antiplatelet therapy was administered.

**Lessons:**

When performing an ultrasound examination of PSA, careful identification of the arterial anatomy, evaluation of blood flow, assessment of surrounding structures, comparison between sides, and correlation with clinical symptoms are crucial to accurately diagnose this rare vascular anomaly.

## 1. Introduction

The sciatic artery is a direct continuation of the internal iliac artery and serves as the main blood supply to the lower limb during fetal development. However, the sciatic artery eventually regresses, leaving behind residual popliteal and fibular arteries. Before the regression of the sciatic artery, the popliteal and fibular arteries establish continuity with the superficial femoral artery (SFA).^[1]^ Persistent sciatic artery (PSA) refers to the persistence of the sciatic artery during fetal development, with an incidence rate of approximately 0.025% to 0.6%, and about 30% of cases are bilateral.^[1,2]^ PSA can be classified into complete and incomplete types based on whether it continues with the popliteal artery or not,^[3]^ with the vast majority of patients belonging to the complete type. Although rare, PSA can still cause serious complications, including arterial aneurysm, distal thromboembolism, and limb-threatening ischemia.^[4,5]^

## 2. Case

A 78-year-old female presented with sudden-onset right lower limb pain, which had been persistent for 5 days. The pain was described as a continuous throbbing sensation, accompanied by a feeling of swelling. The skin temperature of the affected limb was lower than normal. The pain worsened with walking and was associated with numbness and sensory loss in the lower limb. Subsequently, the patient developed difficulty in moving the right lower limb. She sought medical attention at a local hospital, where a Doppler ultrasound revealed thromboembolism in the lower limb arteries.

The patient is generally in good health with no history of diabetes, hypertension, medication or food allergies, surgical history, major illnesses, or cardiac arrhythmias, such as atrial fibrillation.

Physical examination revealed swelling of the right lower limb with slightly increased tissue tension. The skin appeared pale, and there was cyanosis in the foot. The temperature of the skin below the knee joint on the right lower limb was decreased. Tenderness was present in the calf, along with superficial varicose veins. A small bruise was observed on the lateral aspect of the knee joint. The dorsalis pedis and posterior tibial arteries were not palpable in the right lower limb, while the femoral and popliteal arteries had detectable pulses. Sensation was absent beyond the ankle on the right side, and ankle movement was impaired. The deep venous patency test (Perthes test) was negative. No obvious abnormalities were noted in the left lower limb.

Blood biochemistry results showed significantly elevated lactate dehydrogenase (610.9), α-hydroxybutyrate dehydrogenase (510.6), creatine kinase (CK = 7328), and CK isoenzyme level (128.3).

**Figure 1.**
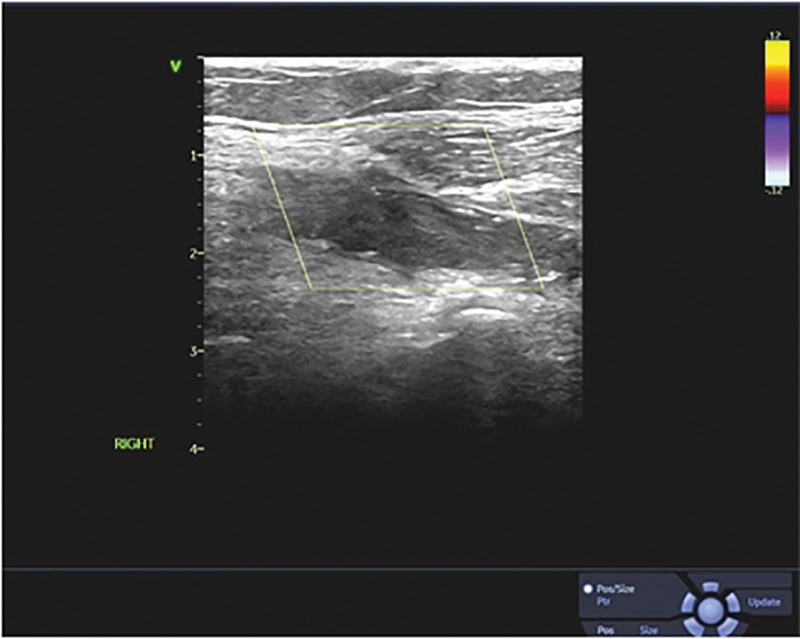
A solid hypoechoic mass within the right popliteal artery as shown on the ultrasound image. Color Doppler flow imaging did not show any significant flow signal within the mass.

Emergency Doppler ultrasonography revealed a thrombus visible in the patient’s right popliteal artery, causing popliteal artery occlusion (Fig. 1).

**Figure 2.**
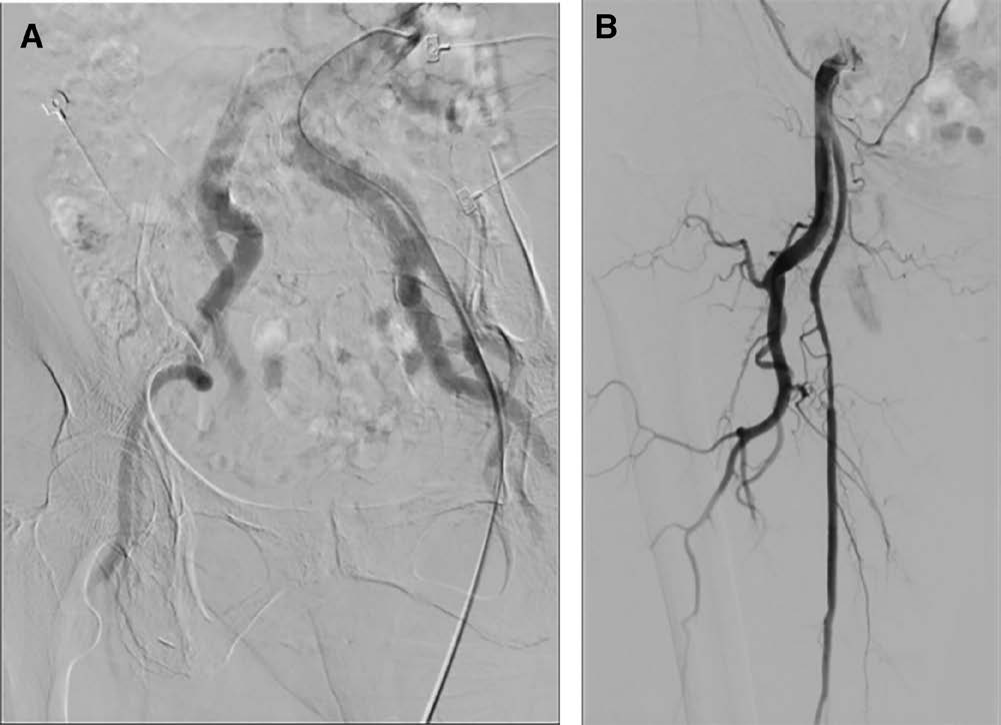
Intraoperative Digital Subtraction Angiography (DSA) images. (A) Bilateral enlarged arteries adjacent to the femoral arteries. (B) Incomplete filling of the superficial femoral artery.

Emergency angiography and treatment: The right SFA did not show any contrast filling, and the catheter and guidewire could not be successfully advanced into the SFA, while the deep femoral artery showed partial filling with a nearby larger diameter vessel visualized (Fig. 2). Subsequently, a single curved catheter was inserted into the femoral artery. Heparin sodium 2000 U and urinary kinase 200,000 U were separately diluted in 50 mL of normal saline in 2 tubes. The drugs were administered for thrombolysis simultaneously at a continuous rate of 4 mL/h via an infusion pump.

**Figure 3.**
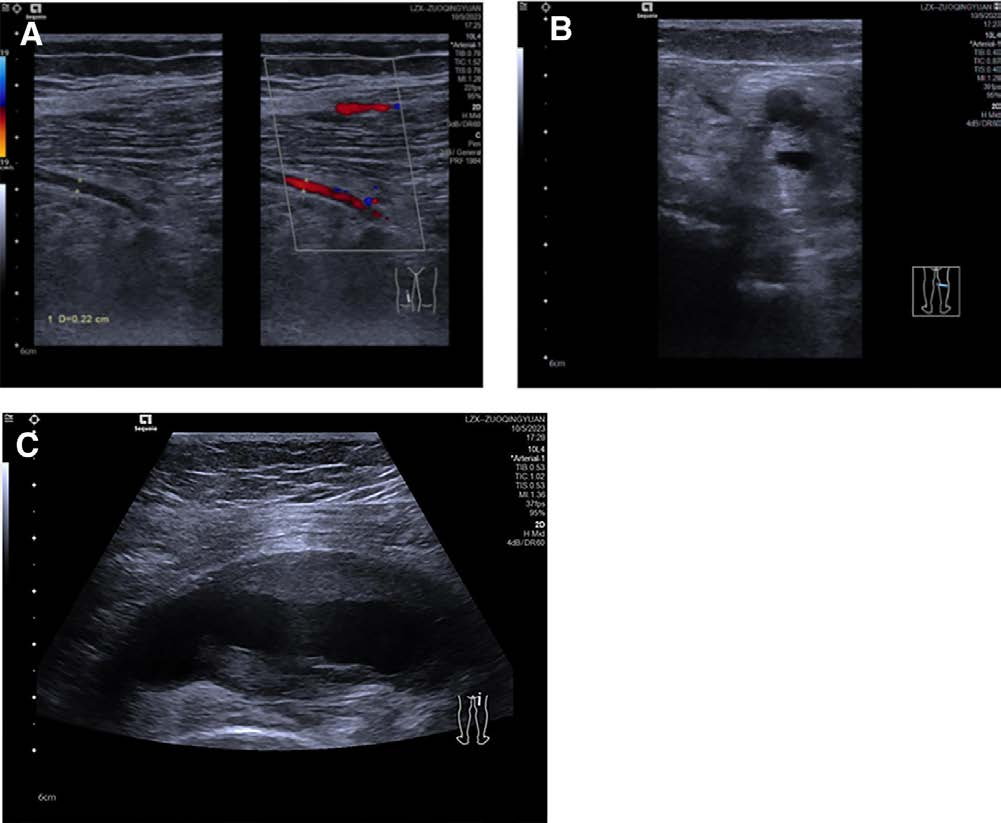
(A) The diameter of the common femoral artery was normal, while the superficial femoral artery gradually narrowed but still had a patent blood flow. (B) A large tubular hypoechoic structure was observed behind the distal end of the superficial femoral artery, which connected to the popliteal artery. (C) Scanning from bottom to top revealed that the large vessel extended upward to the level of the ischial tuberosity and into the pelvic cavity, with a tumor-like dilation at its proximal end and an intraluminal thrombus.

Postoperative Doppler ultrasound examination: Lower limb arterial variation, bilateral PSA, true arterial aneurysm with intraluminal thrombus in the right PSA, and poor development of bilateral superficial femoral arteries (Fig. 3).

**Figure 4.**
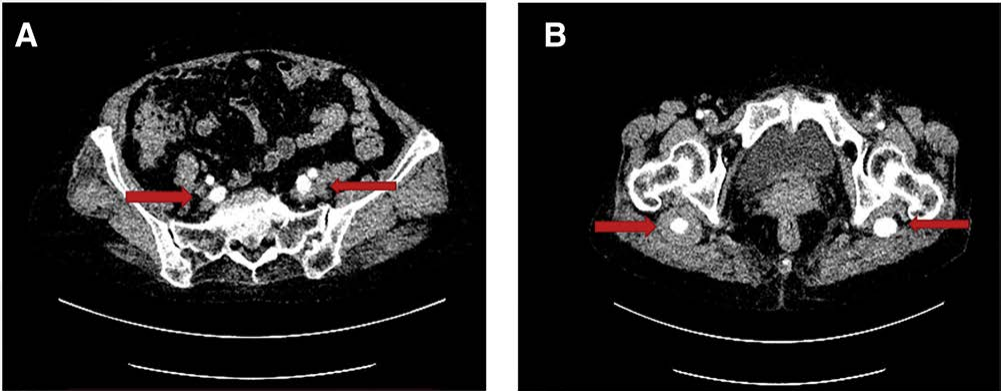
(A) Marked dilation of bilateral internal iliac arteries shown by red arrows. (B) Aneurysmal dilation of bilateral sciatic arteries, with an intraluminal thrombus in the right side shown by red arrows.

**Figure 5.**
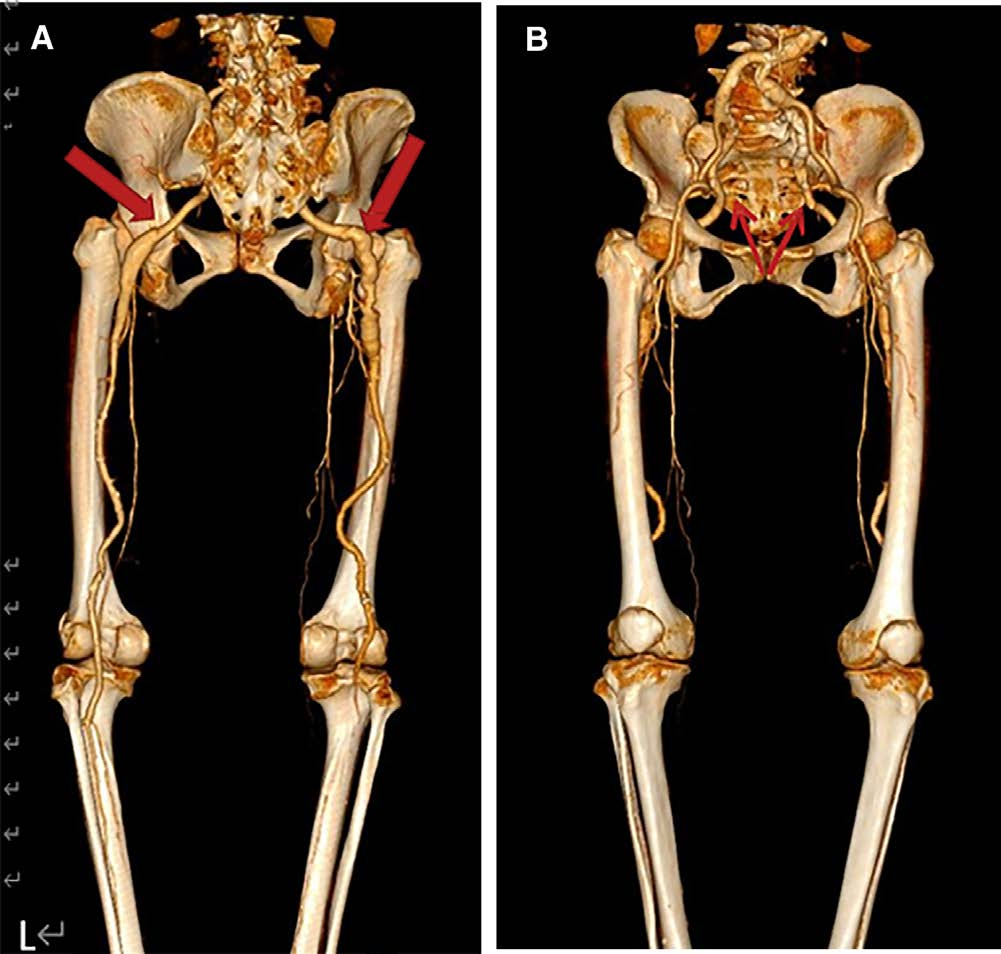
(A and B) The computed tomography angiogram of the lower extremity’s runoff shows bilateral internal iliac arteries widening, extending downward to the popliteal arteries. There is a thin development of bilateral superficial femoral arteries. True arterial aneurysms have formed in the bilateral sciatic arteries, with an intraluminal thrombus present in the right sciatic artery. The course of the sciatic artery is indicated by red arrows.

Contrast-enhanced computed tomography (CT) and CT angiography findings: bilateral PSA and bilateral aneurysm with thromboembolism of the right lower extremity (Figs. 4 and 5).

In the Leriche classification of acute lower limb ischemia, the patient presents with pain, tenderness, localized superficial vein varicosities, pale skin, cyanosis, and decreased skin temperature, indicating moderate ischemic symptoms consistent with class IIb. Despite the patient’s general condition being stable, this suggests the presence of moderate ischemic symptoms in the patient. While not in immediate danger, prompt evaluation and treatment are still necessary to prevent disease progression. Following emergency thrombolysis, the blood flow to the patient’s lower limbs has been restored, and the symptoms of lower limb embolism have disappeared. After evaluating multiple factors including the patient’s age and physical condition, we have determined that it is unnecessary to subject the patient to the risks of surgical ligation for the PSA artery aneurysm. Following an explanation of the relevant treatment options and their associated risks to the patient and their family, upon the patient’s request, discharge was granted after several days of heparin therapy. The patient was advised to improve their lifestyle habits, adhere to regular antiplatelet medication (daily doses: aspirin, 100 mg; clopidogrel, 75 mg; atorvastatin calcium tablets, 20 mg; and pantoprazole, 40 mg), and attend scheduled follow-up appointments.

## 3. Discussion

PSA has various types, which can be classified according to the integrity of the PSA and the status of the SFA. The Pillet-Gauffre classification system^[6,7]^ divides PSA into 12 types based on these factors, as shown in Figure 1. In addition, Ahn et al^[8]^ proposed a new classification system in 2016, called the ccxAhn-min classification system, which adds the presence of an arterial aneurysm to the Pillet-Gauffre classification system. The case that we reported involves the presence of the SFA, but it does not reach the level of the popliteal artery and, thus, belongs to type 2a in the Pillet-Gauffre classification system and type IIIa in the ccxAhn-min classification system.

Most cases of PSA are incidentally diagnosed due to the corresponding symptoms caused by its complications, such as arterial aneurysm and distal thromboembolism.^[4]^ The formation of arterial aneurysm is believed to be caused by repeated trauma to the buttocks due to the compression of the sacrospinous ligament, piriformis muscle, and hip joint, as well as the frequent stretching of the arterial wall elastic fibers during hip joint flexion.^[9]^ Arterial aneurysm can then compress the sciatic nerve, leading to corresponding symptoms and signs.^[10]^ The formation of intraluminal thrombosis in the vascular tumor and the stenosis or occlusion of the distal artery are the most common reasons for the discovery of PSA.

Ultrasound is a low-cost, easy-to-operate, real-time, safe, and reliable imaging method.^[11]^ In emergency situations of acute thrombosis, ultrasound can provide immediate, noninvasive vascular imaging information through real-time imaging and Doppler technology, helping doctors quickly and accurately determine the location and severity of the thrombosis. Combined with comprehensive information such as medical history, physical examination, and CK spectrum, the severity of the thrombosis can be further evaluated, thereby determining whether emergency thrombolysis treatment or other emergency treatment measures are needed. After emergency thrombolysis treatment, ultrasound can also be used to detect the recovery of blood flow and tissue perfusion, helping to evaluate the treatment effect and predict the prognosis. Ultrasound contrast and elastography technology can also be used to evaluate the stability of plaques and thrombi and select effective intervention measures to avoid the recurrence of thrombosis.^[12]^ Compared with contrast-enhanced CT and CTA, ultrasound can observe the location of intravascular thrombosis and evaluate the severity of thrombosis in a comprehensive and 3-dimensional manner. CTA is the gold standard for diagnosing vascular variations, but it is time-consuming and may cause contrast agent allergies, so it is not the first choice for emergency critically ill patients.

The treatment for PSA can be divided into treating symptomatic aneurysms and managing acute limb ischemia secondary to thrombotic aneurysms and/or distal embolization. Due to the variability in anatomical structures and the range of symptoms, different treatment strategies are often matched accordingly. According to the Rutherford classification^[13]^ for acute limb ischemia, catheter-directed thrombolysis (CDT) is typically considered a feasible (Rutherford I) or mildly threatening (Rutherford IIa) initial treatment strategy for ALI.^[14]^ In the face of complex anatomical structures and unclear thrombosis, CDT can also be considered an urgently effective treatment method. However, there are also potential complications during the CDT process, such as distal embolism and bleeding,^[15,16]^ and postoperatively, there is still a risk of recurrent limb thrombosis. Therefore, the definitive treatment for this patient should be surgical femoropopliteal bypass and ligation of the PSA aneurysm.^[17]^ Due to the incomplete development of SFA in this case, the recommended definitive treatment plan for the patient involves SFA revascularization through surgical bypass. To prevent the recurrence of arterial embolism, the aneurysm should be treated first, requiring either surgical or endovascular excision, and elimination through coil embolization or stent graft placement.

The study has several limitations. First, the patient did not adhere to the scheduled follow-up appointments, resulting in a lack of information on the thrombus resolution postantiplatelet therapy. Second, the use of contrast-enhanced ultrasound imaging was not employed to further assess the filling status of the femoral artery and SFA in the patient.

## 4. Conclusion

For clinical practitioners, the etiological diagnosis of lower extremity arterial ischemia remains a significant challenge. In the case of acute lower extremity arterial thrombosis, emergency thrombolysis is one of the treatment options for acute limb ischemia, aiding in risk mitigation and tracking the origin of the thrombus. Ultrasonography facilitates real-time monitoring of abnormalities, aiding in the exploration of underlying causes. In the management of peripheral arterial disease, while advanced age and frailty should not automatically preclude treatment, careful consideration of risks and benefits is essential. In elderly patients without evident ischemic symptoms, who experience sudden resolution of symptoms following acute limb ischemia upon reperfusion, surgical intervention may not be necessary. Long-term antiplatelet therapy and regular follow-up are recommended for such individuals.

## Author contributions

**Conceptualization:** Yi Mao.

**Data curation:** Yi Mao, Zhi-Xing Liu.

**Visualization:** Yi Mao, Li Chen, Zhi-Xing Liu.

**Writing – original draft:** Yi Mao.

**Writing – review & editing:** Yi Mao, Li Chen, Zhi-Xing Liu.

**Validation:** Zhi-Xing Liu.
